# The Combined Effects of Moss-Dominated Biocrusts and Vegetation on Erosion and Soil Moisture and Implications for Disturbance on the Loess Plateau, China

**DOI:** 10.1371/journal.pone.0127394

**Published:** 2015-05-20

**Authors:** Chongfeng Bu, Shufang Wu, Fengpeng Han, Yongsheng Yang, Jie Meng

**Affiliations:** 1 Northwest A & F University, State Key Laboratory of Soil Erosion and Dryland Farming on the Loess Plateau, Yangling, Shaanxi, China; 2 Institute of Soil and Water Conservation, Chinese Academy of Sciences and Ministry of Water Resources, Yangling, Shaanxi, China; 3 College of Water Resources and Architectural Engineering, Northwest A & F University, Yangling, Shaanxi, China; Beijing Normal University, CHINA

## Abstract

Biological soil crusts (BSCs, or biocrusts) have important positive ecological functions such as erosion control and soil fertility improvement, and they may also have negative effects on soil moisture in some cases. Simultaneous discussions of the two-sided impacts of BSCs are key to the rational use of this resource. This study focused on the contribution of BSCs while combining with specific types of vegetation to erosion reduction and their effects on soil moisture, and it addressed the feasibility of removal or raking disturbance. Twelve plots measuring 4 m × 2 m and six treatments (two plots for each) were established on a 15° slope in a small watershed in the Loess Plateau using BSCs, bare land (as a control, BL), *Stipa bungeana* Trin. (STBU), *Caragana korshinskii* Kom. (CAKO), STBU planted with BSCs (STBU+BSCs) and CAKO planted with BSCs (CAKO+BSCs). The runoff, soil loss and soil moisture to a depth of 3 m were measured throughout the rainy season (from June to September) of 2010. The results showed that BSCs significantly reduced runoff by 37.3% and soil loss by 81.0% and increased infiltration by 12.4% in comparison with BL. However, when combined with STBU or CAKO, BSCs only made negligible contributions to erosion control (a runoff reduction of 7.4% and 5.7% and a soil loss reduction of 0.7% and 0.3%). Generally, the soil moisture of the vegetation plots was lower in the upper layer than that of the BL plots, although when accompanied with a higher amount of infiltration, this soil moisture consumption phenomenon was much clearer when combining vegetation with BSCs. Because of the trivial contributions from BSCs to erosion control and the remaining exacerbated consumption of soil water, moderate disturbance by BSCs should be considered in plots with adequate vegetation cover to improve soil moisture levels without a significant erosion increase, which was implied to be necessary and feasible.

## Introduction

Biological soil crusts (BSCs, or biocrusts) are complex communities containing bacteria, fungi, lichens, and moss cemented with soil particles. These layers represent the typical ground cover in arid and semi-arid regions and have important ecological functions [1,2,3]. The presence of BSCs significantly affects the physical structure, chemical properties, and biological characteristics of soil. BSCs also impact rainfall infiltration, soil water evaporation, soil erosion, geochemical cycling and species diversity. The developmental and ecological functions of BSCs vary across spatial and temporal scales, as shown in the vast majority of studies in warm and cool deserts at 30°-60° in the middle latitudes [4]. It is widely believed that BSCs fertilize the soil [5,6], increase soil stability [7], and reduce the extent of water or wind erosion [8,9]. However, under different conditions, researchers have reported positive, negative, and neutral relations between BSCs and rainfall infiltration, water evaporation, and vegetation succession [10]. Many studies have shown that BSCs influence the redistribution of rainfall in soil profiles over the long term but also increase the consumption of soil water and reduce soil moisture, which ultimately hinders plant growth [11,12,13]. Therefore, comprehensive investigations on the effects of BSCs on hydrology, soil erosion, and rainfall redistribution are highly valuable for both theoretical and practical purposes, with the aim of understanding a specific condition or region. Fully understanding the positive significance and negative effects of BSCs is the key to balancing between protection and disturbance and to realizing the efficient utilization of these resources.

The Loess Plateau in China is characterized by its unique geomorphology and soil type. Drought and severe soil and water loss are considered to be major environmental issues in this area. The implementation of the “Grains for Green Project” since 1999 has generated substantial environmental benefits and has greatly improved the degraded ecosystems in the Loess Plateau [14]. BSCs are indicative of ecological recovery and are widely distributed. They have a surface coverage of >70% in certain areas of the Loess Plateau [15]. BSCs may affect water erosion and soil moisture in the Loess Plateau region in various ways because of the unique types of BSCs, vegetation communities and soil. Preliminary research has indicated that moss crust covers a large proportion of the Loess Plateau, where it is known to improve soil fertility [16,17], increase infiltration, reduce runoff and greatly decrease soil erosion [18]. Unfortunately, little is known about the effects of BSCs on soil moisture in preventing soil erosion especially in combination with different types of vegetation. The present knowledge regarding the effects of BSCs in the Loess Plateau of China is not sufficient to provide theoretical support for the effective practical management of BSCs.

Based on previous studies, we hypothesized that BSCs would prevent erosion and increase the consumption of soil water on a hillslope on the Loess Plateau. An appropriate balance must therefore be established between the protection and disturbance of BSCs to optimize their positive effects and minimize their negative influences. The contribution of BSCs to erosion prevention is most likely small if they are combined with various types of vegetation. At the same time, they continue to contribute to soil water consumption. Therefore, moderate disturbance measures such as removal or raking may be necessary to improve soil water conditions while maintaining soil erosion control. These actions would positively benefit subsequent vegetation growth. Based on this hypothesis, we established 12 plots with various combinations of two dominant native plant species and BSCs and measured the runoff, soil loss, and soil moisture in 3 m soil profiles within 24 h of rainfall events. These data were used to address the following questions: (i) Do BSCs reduce soil erosion on slopes? (ii) What is the contribution of BSCs to erosion reduction if they are combined with specific types of vegetation? (iii) What are the effects of BSCs on soil moisture at different depths? (iv) Do BSCs have different effects on soil moisture in the presence of various types of vegetation? (v) Is it theoretically possible to improve soil water conditions by introducing suitable disturbance measures while avoiding soil erosion?

## Materials and Methods

### Study site

**Fig 1:**
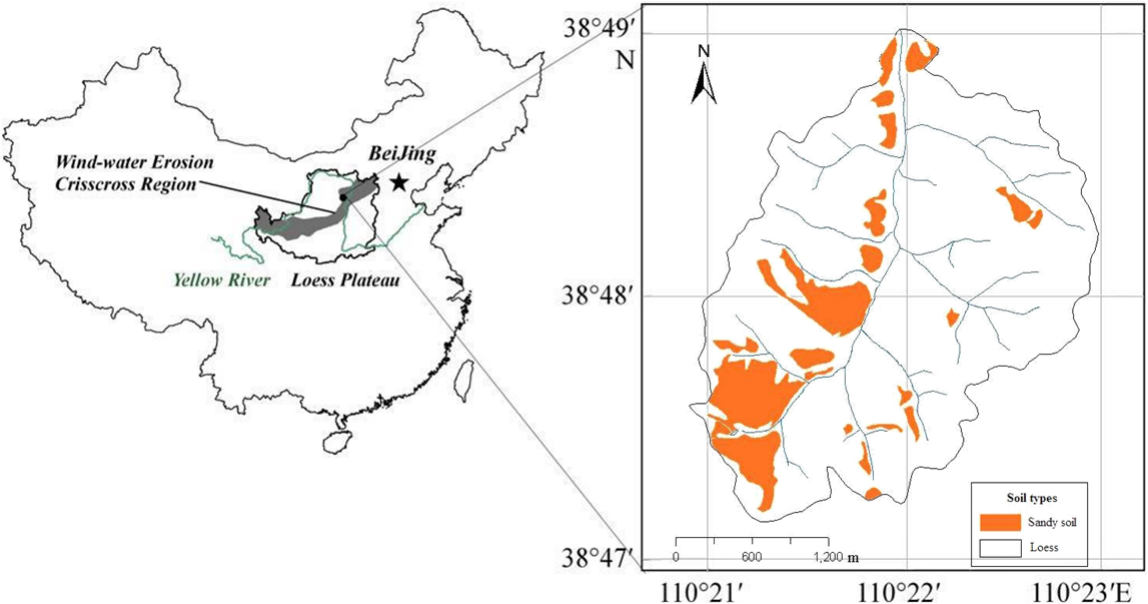
The location of the watershed study site (orange and white represent sandy and loess soils, respectively) and the wind-water erosion crisscross region (gray area) in the Loess Plateau of China.

This study was conducted in the Liudaogou watershed in the western region of Shenmu County, Shaanxi Province, China (110°21′ to 110°23′E, 38°46′ to 38°51′N). The altitude ranges from 1094 to 1274 m, and the watershed area is 6.89 km^2^. The primary gully (length = 4.21 km), which is the second tributary of the Kuye River, runs from north to south. The Liudaogou watershed (Fig 1) is located in the Loess Plateau on the margin of the Mu Us Desert. The vegetation type in the watershed belongs to a transitional zone from forest steppe to dry steppe. The watershed is subject to wind and water crisscross erosion of the loess soils in hilly areas, and the annual soil erosion rate is approximately 10,000 t/km^2^ [19]. The climate is classified as sub-arid temperate zone. Spring and winter are characterized by less rain and more wind, and there is more rain in the summer and autumn, with frequent rainstorms and severe water erosion. The mean annual temperature is 8.4°C. The prevailing wind is from the northwest, and the mean annual wind speed is 2.2 m/s. The mean annual precipitation is approximately 400 mm, and the precipitation from June to September represents approximately 70–80% of the annual precipitation. Shrub vegetation is predominant and includes *Caragana korshinskii* Kom. (CAKO, a typical native shrub species), *Stipa bungeana* Trin. (STBU, a typical native grass species), *Medicago sativa* Linn., *Lespedeza dahurica* (Laxim.) Schindl., *Heteropappus altaicus* (Willd.) Novopokr., and various Asteraceae species. This study was approved by Northwest A & F University.

### Methods

#### Experimental design

**Fig 2:**
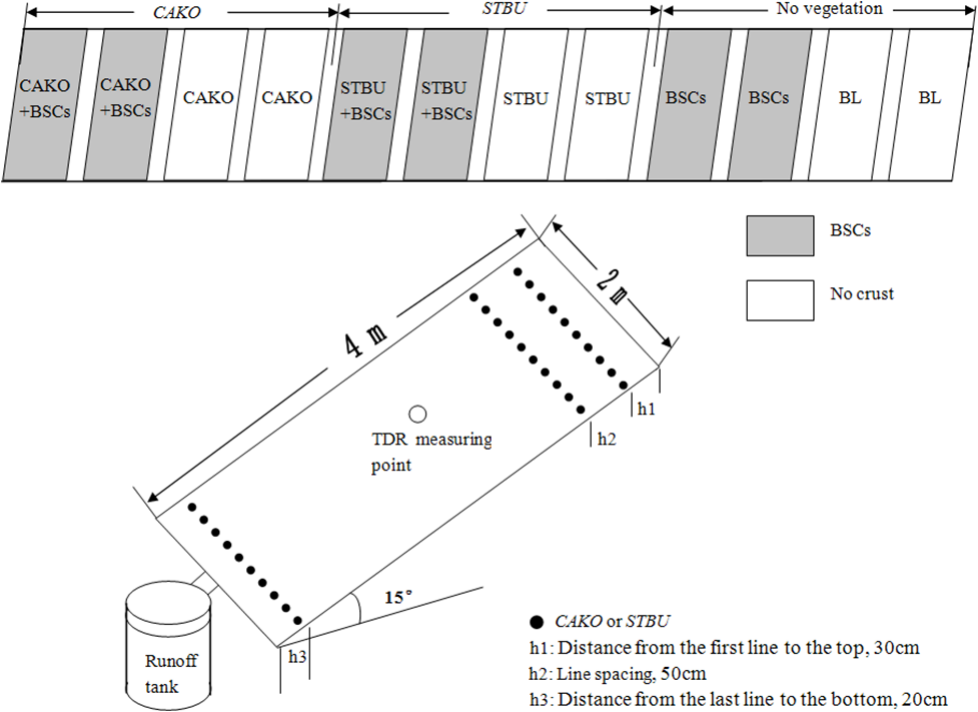
Experimental configuration and measured locations for each plot.

In 2008, twelve runoff plots measuring 4 m × 2 m were established on a 15° middle slope that faces northeast and were situated in the central of the catchment. The plots were located in the middle of the partially shaded slope and were arranged in a horizontal line (Fig 2). In the third year (2010) of the study, we recorded the runoff, soil loss and soil moisture in the 3 m soil profiles of these plots within 24 h of rainfall events. Collection tanks 30 cm in diameter and 100 cm in height were placed at the lower side of each plot to collect runoff and soil loss during each rainfall event. The 0–20 cm soil profile was composed of 6.33% clay (<0.002 mm), 22.11% silt (0.002–0.02 mm), and 71.57% sand (>0.02 mm), and the bulk density and saturated water content of the soil was 1.39 g/cm^3^ and 35.11%, respectively [16], which indicates the basic physical properties of all plots due to the relative homogeneity of Loess soil in profile [20,21]. A total of six treatments were created (Table 1), and two plots were established for each treatment. For the runoff and sediment, eighteen replicates from two plots at each treatment after 9 runoff-producing rainfall events were used for statistical analysis. All experimental plots are regulated by the Institute of Soil and Water Conservation, Chinese Academy of Sciences and Ministry of Water Resources. The individual in this manuscript has given written informed consent (as outlined in the PLOS consent form) to publish these case details.

**Table 1:** Vegetation and BSCs parameters in the experimental plots.

Treatment^1^	BSCs depth^2^ (mm)	Shear strength^3^ (kPa)	BSCs cover (%)	Vegetation cover (%)
BL	-	21.2±6.6	0	0
BSCs	10.2±1.9	36.2±7.3	95	0
STBU	-	27.9±6.7	0	45
STBU + BSCs	10.4±1.7	34.4±8.5	95	45
CAKO	-	27.4±10.0	0	55
CAKO + BSCs	11.5±1.0	41.2±12.3	95	55

^1^BL, bare land; BSCs, biological soil crusts; STBU, *Stipa bungeana* Trin.; and CAKO, *Caragana korshinskii* Kom.

^2^ Measured using calipers. Mean ± SE (n = 12)

^3^ Measured using a pocket shear gauge on relatively dry surface soil. Mean ± SE (n = 20).

For the CAKO plots, seeds were drill-sown approximately 3 cm depth during May 2008 with 50 cm of line spacing in four bare plots and thinned out to a 30 cm row spacing after emergence. For the STBU plots, seedlings in the watershed were transplanted into the four bare plots using 30 cm row spacing and 50 cm line spacing. The distance from the first sowing line in the plot to the top was 30 cm, and the distance from the last line in the plot to the bottom was 20 cm (Fig 2).

BSCs were developed using the “mosaic” method [2, 22], i.e., patches of BSCs were collected from the sampling area in the watershed and reassembled in the experimental plots to create the combined plots (i.e., STBU+BSCs and CAKO+BSCs) in July 2008. Only well-developed moss crusts were selected (with a BSCS thickness of approximately 10 mm and coverage >90%). The collected crusts, together with the substrate (the total thickness was 2–3 cm), were then transported and carefully placed manually into the plots in an irregular pattern. After three rainy seasons, the small gaps that were created while transplanting the crusts closed naturally, and the moss crust developed evenly and grew well in comparison with natural conditions. The coverage and thickness of the BSCs in each plot were measured and are listed in Table 1. The dominant species of the local moss crust included *Bryum argenteum* Hedw. of the Bryaceae and *Didymodon constrictus* (Mitt.) Saito of the Pottiaceae [23]. Two bare plots without vegetation or BSCs (BL, bare land plots) were used as controls. The weeds in all the plots were removed carefully at regular intervals without BSCs or vegetation damage. The BSCs pavement processes and a panoramic view of the experimental plots is shown in S1 Fig. When runoff, infiltration, and soil moisture measurements were performed in 2010, the BSC coverage was paved artificially by approximately 95%, and the coverage levels in the CAKO and STBU plots were 55% and 45%, respectively.

#### Monitoring methods

Time domain reflectometry (TDR, IMKO Company, Germany) with intelligent microelements was used to monitor the variation in the soil moisture at depths of 0 to 300 cm (in 10 cm layers) in the middle of each plot (Fig 2) in June (early in the rainy season) and September (at the end of the rainy season) of 2010, and one probe pipe was installed before BSC transplant or vegetation planting in the middle of each plot. The values reading are obtained automatically through a probe-inserted pipe during the measurement. Ten soil moisture values from two plots within each 50 cm profile (10 cm interval) were considered as replicates of each treatment for statistical analysis. The amount of rainfall was measured using a standard rain gauge placed near the experimental plots. The runoff and eroded soil were collected using collection tanks at the lower border of each plot (Fig 2) and were measured within 24 h of each rainfall event.

### Data analysis

We calculated the following indices based on field measurements:

$$RC=R\;/\;R_{a}$$ (1)

$$IC=I\;/\;R_{a}$$ (2)

$$ERR=\left(1-R_{t}\;/\;R_{b}\right)\times 100\left(\%\right)$$ (3)

Where RC is the Runoff coefficient, R is runoff, R_a_ is rainfall, IC is the Infiltration coefficient, I is infiltration, ERR is the Efficiency of runoff reduction, R_t_ is the runoff of a treatment, and R_b_ is the runoff of BL.

This index represents the efficiency of a treatment in reducing runoff in comparison with the BL plot.

$$CBRR=\left(\left(1-R_{tb}\;/\;R_{b}\right)-\left(1-R_{twb}\;/\;R_{b}\right)\right)\times 100=E_{CTRR}-E_{VTRR}\left(\%\right)$$ (4)

Where CBRR is the Contribution of BSC to runoff reduction, R_tb_ is the runoff of the treatment with BSC, R_b_ is the runoff of BL, R_twb_ is the runoff from the treatment without BSC, E_CTRR_ is the Efficiency of the combined treatment in runoff reduction, and E_VTRR_ is the Efficiency of the vegetation-only treatment in runoff reduction.

This index describes the BSC contribution to the runoff reduction when combined with vegetation.

$$EII=\left(I_{t}\;/\;I_{b}-1\right)\times 100\left(\%\right)$$ (5)

Where EII is the Efficiency of increased infiltration, I_t_ is the infiltration of a treatment, and I_b_ is the infiltration of BL.

This index represents the efficiency of a treatment in increasing infiltration in comparison with the BL plot.

$$CBII=\left(\left(1-I_{tb}\;/\;I_{b}\right)-\left(1-I_{twb}\;/\;I_{b}\right)\right)\times 100=E_{CTII}-E_{VTII}\left(\%\right)$$ (6)

Where CBII is the Contribution of BSC to increased infiltration, I_tb_ is the infiltration of the treatment with BSC, I_b_ is the infiltration of BL, I_twb_ is the infiltration of the treatment without BSC, E_CTII_ is the Efficiency of the combined treatment in increasing infiltration, E_VTII_ is the Efficiency of the vegetation-only treatment in increasing infiltration.

This index represents the BSC contribution to the increase in infiltration when combined with vegetation.

$$ESR=\left(1-S_{t}\;/\;S_{b}\right)\times 100\left(\%\right)$$ (7)

Where ESR is the Efficiency in soil loss reduction, S_t_ is the soil loss in a treatment, and S_b_ is the soil loss in BL.

This index represents the efficiency of a treatment in reducing soil loss in comparison with the BL plot.

$$CBSR=\left(\left(1-S_{tb}\;/\;S_{b}\right)-\left(1-S_{twb}\;/\;S_{b}\right)\right)\times 100=E_{CTRR}-E_{VTRR}\left(\%\right)$$ (8)

Where CBSR is the Contribution of BSCs to soil loss reduction, S_tb_ is the soil loss of the treatment with BSC, S_b_ is the soil loss in BL, S_twb_ is the soil loss of the treatment without BSC, E_CTSR_ is the Efficiency of the combined treatment in soil loss reduction, and E_VTSR_ is the Efficiency of the vegetation-only treatment in soil loss reduction.

This index describes the BSC contribution to the reduction in soil loss when combined with vegetation.

The study data were analyzed with Microsoft Excel 2007. Under the nine runoff-producing rainfall events, eighteen measurements of runoff and sediment for each treatment with two replicate plots were expressed as the Mean ± SE. Ten soil moisture values from two plots within each 50 cm profile were presented as the Mean ± SE. All measured values of the same index for each treatment were compared with the difference between treatments using a one-way ANOVA and LSD tests in SPSS software (SPSS Inc. Chicago, IL, USA).

## Results

### Effects of BSCs and vegetation on runoff and infiltration

**Fig 3:**
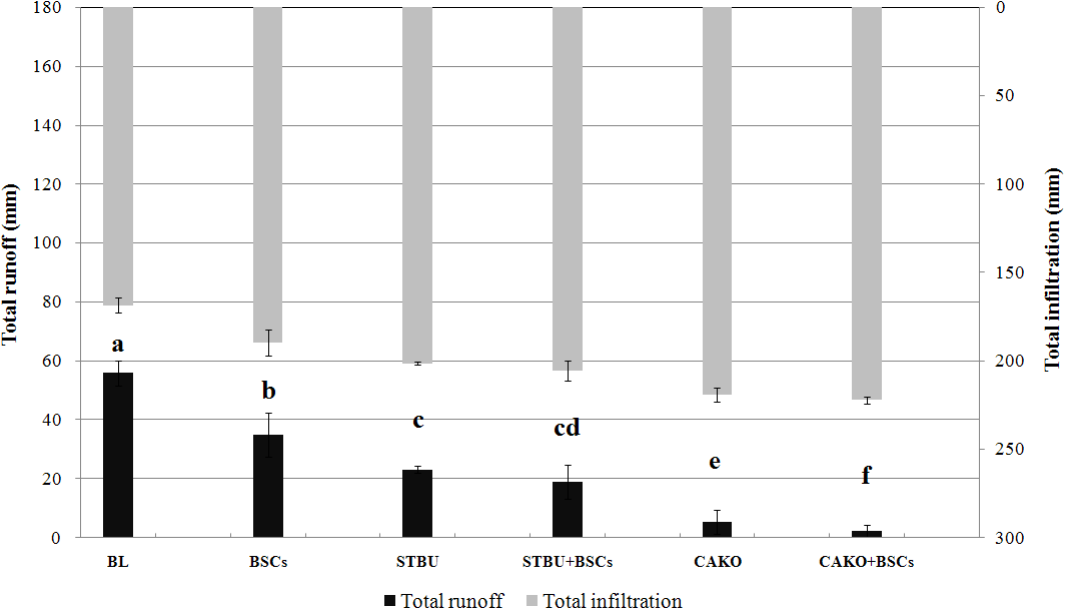
The runoff and infiltration for the different treatments after the nine rainfall events. Note: The values were presented as the Mean ± SE, different letters indicate significant differences at *P*< 0.05. Abbreviations: BL, bare land; BSCs, biological soil crusts; STBU, *Stipa bungeana* Trin.; CAKO, *Caragana korshinskii* Kom.

Nine rainfall events totaling 224.5 mm occurred during the study. The total runoff and infiltration differed significantly among treatments except STBU and STBU+BSCs (*p*<0.05) (Fig 3). The runoff decreased in the following order: CAKO+BSCs > CAKO > STBU+BSCs > STBU > BSCs > BL. The runoff from the BSCs plots and the BSCs plus vegetation plots was significantly lower than that of the control (BL). By contrast, the infiltration increased as the runoff decreased. Greater runoff reduction was observed in the combined BSC treatments with vegetation.

In compared with BL, the effects of BSCs alone on the runoff reduction and infiltration increase were 37.3% and 12.4%, respectively. These effects were smaller than the corresponding effects of the STBU treatment (58.5% and 19.4%, respectively) or the CAKO treatment (90.1% and 29.9%, respectively) (Table 2). The runoff and infiltration coefficients showed the same trend as the runoff and infiltration, and there were similar significant differences among the treatments (*p*<0.05). The effects of vegetation on runoff and infiltration were substantially greater than the effects attributed to BSCs alone. The effects were greatest when STBU or CAKO was combined with BSCs. For example, the STBU+BSCs treatment reduced runoff by 65.9% and increased infiltration by 21.9%. The CAKO+BSCs treatment reduced runoff by 95.8% and increased infiltration by 31.8%. In the STBU+BSCs treatment, the BSCs reduced runoff by 7.4% and increased infiltration by 2.5%. In the CAKO+BSCs treatment, the BSCs reduced runoff by 5.7% and increased infiltration by 1.9%, which was smaller than the effect of the BSCs treatment (*p*<0.05). This finding suggests that although BSCs greatly reduced the runoff and increased infiltration relative to BL, their effects were smaller than those found if BSCs were combined with vegetation (no significant difference was observed between CAKO and STBU: *p*>0.05). Thus, vegetation played an important role in controlling runoff and promoting infiltration, whereas BSCs under vegetation contributed very little although BSCs alone had a substantial effect on runoff.

**Table 2:** Runoff and infiltration after nine rainfall events (cumulative rainfall = 224.5 mm).

Treatment^1^	Runoff coefficient (RC)	Infiltration coefficient (IC)	Efficiency in runoff reduction (ERR, %)	Efficiency in increasing infiltration (EII, %)	Contribution of BSC to runoff reduction (CBRR, %)	Contribution of BSC to increasing infiltration (CBII, %)
BL	0.25±0.02^a^	0.75±0.02^f^	0.0^f^	0.0^f^	37.3±13.2^a^	12.4±4.4^a^
BSCs	0.16±0.03^b^	0.84±0.03^e^	37.3±13.2^e^	12.4±4.4^e^		
STBU	0.10±0.00^c^	0.90±0.00^cd^	58.5±1.9^d^	19.4±0.6^d^	7.4±8.6^b^	2.5±2.9^b^
STBU +BSCs	0.08±0.03^cd^	0.92±0.03^c^	65.9±5.5^c^	21.9±3.5^c^		
CAKO	0.02±0.02^e^	0.98±0.02^b^	90.1±7.4^b^	29.9±2.4^b^	5.7±3.9^c^	1.9±1.3^b^
CAKO + BSCs	0.01±0.01^f^	0.99±0.01^a^	95.8±3.4^a^	31.8±1.2^a^		

Note: Different superscript letters in the same column indicate significant differences at *P*< 0.05

^1^BL, bare land; BSCs, biological soil crusts; STBU, *Stipa bungeana* Trin.; CAKO, *Caragana korshinskii* Kom.

### Effects of BSCs and vegetation on erosion

During this study, the relation between runoff accumulation and soil loss in each treatment after the nine rainfall events showed that soil loss could be approximated by an exponential model of accumulated runoff (*y* = 3.752e^0.111*x*^, R^2^ = 0.978, where *y* is the cumulative soil loss and *x* is the cumulative runoff). The intensity of erosion increased sharply as a function of the runoff intensity.

**Fig 4:**
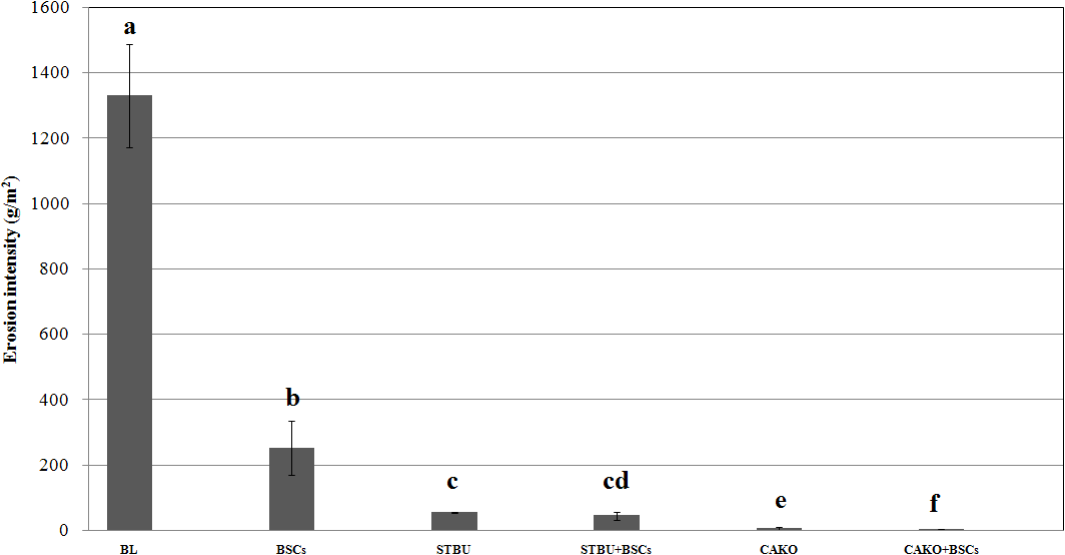
The erosion intensity for the different treatments after the nine rainfall events. Note: The values were presented as the Mean ± SE, different letters indicate significant differences at *P*< 0.05. Abbreviations: BL, bare land; BSCs, biological soil crusts; STBU, *Stipa bungeana* Trin.; CAKO, *Caragana korshinskii* Kom.

Both BSCs and vegetation significantly reduced the extent of erosion (*p*<0.05) (Fig 4, Table 3). The erosion reduction efficiencies of different treatments were as follows: CAKO+BSCs (99.8%) and CAKO (99.5%) > STBU+BSCs (96.6%) and STBU (95.9%) > BSCs (81.0%). There were no significant differences in the erosion reduction efficiencies between the STBU and STBU+BSCs treatments or between the CAKO and CAKO+BSCs treatments (*p*>0.05). On average, BSCs alone reduced the soil loss by 81.0%, with a corresponding contribution to erosion reduction of 81.0%. The contribution of BSCs in the combined treatments to the reduced soil loss was only 0.7% in the STBU+BSCs treatment and 0.3% in the CAKO+BSCs treatment. This finding indicates that vegetation was responsible for most of the erosion reduction in the BSCs plus vegetation treatment. Based on these results and the analysis of runoff and infiltration described above, it can be concluded that the BSC contribution to runoff reduction or soil loss was not substantial when adequate vegetation was present.

**Table 3:** Soil erosion in different treatments after the rainfall events.

Treatment^1^	Total erosion density (g/m^2^)	^2^Efficiency in soil loss reduction (ESR, %)	^3^Contribution of BSC to soil loss reduction (CBSR, %)
BL	1329.1±158.4^a^	0.0^d^	81.0±6.3^a^
BSCs	252.8±83.5^b^	81.0±5.3^c^	
STBU	55.1±2.0^c^	95.9±0.2^b^	0.7±1.1^b^
STBU + BSCs	45.4±12.0^cd^	96.6±0.9^b^	
CAKO	6.6±4.1^e^	99.5±0.3^a^	0.3±0.2^b^
CAKO + BSCs	3.3±1.5^f^	99.8±0.1^a^	

Note: Different superscript letters in the same column indicate significant differences at *P*< 0.05

^1^BL, bare land; BSCs, biological soil crusts; STBU, *Stipa bungeana* Trin.; CAKO, *Caragana korshinskii* Kom.

### Effects of BSCs and vegetation on soil moisture

**Fig 5:**
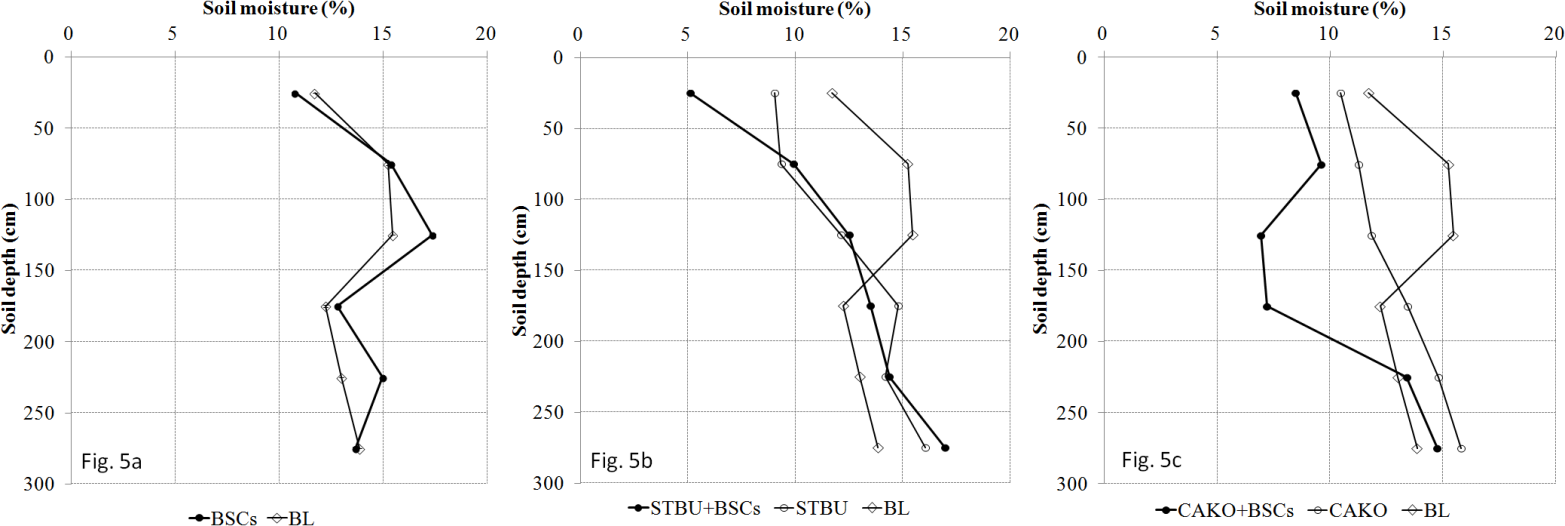
The distribution of soil moisture in the 0–300 cm soil profile at the beginning of the rainy season (20 June 2010). Abbreviations: BL, bare land; BSCs, biological soil crusts; STBU, *Stipa bungeana* Trin.; CAKO, *Caragana korshinskii* Kom.

**Fig 6:**
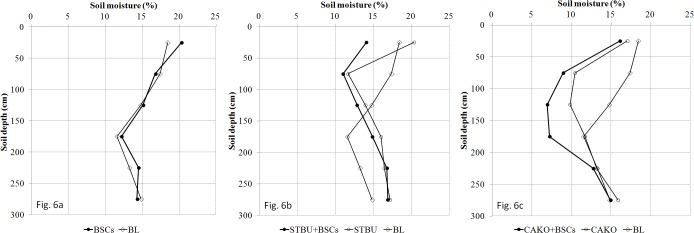
The distribution of soil moisture in the 0–300 cm soil profile at the end of the rainy season (21 September 2010). Abbreviations: BL, bare land; BSCs, biological soil crusts; STBU, *Stipa bungeana* Trin.; CAKO, *Caragana korshinskii* Kom.

When compared with the same treatment in June and September (Figs 5A and 6A, Figs 5B and 6B, Figs 5C and 6C), as a whole, each treatment had higher soil moisture levels at the end of the rainy season than at the beginning in the top 100 cm of soil. At the beginning of the rainy season, the precipitation and soil water consumption were low, and the soil moisture in the six treatment plots was less than or close to 15%. In comparison with the BL plots, the soil moisture of the covered plots (with BSCs, vegetation, or both) was lower in the upper layer (0–150 cm) and higher in the lower layer (150–300 cm) (*p*<0.05, except for 50–100 cm and 100–150 cm in the BSC plots; Tables 4 and 5).

**Table 4:** The soil water content in each treatment at 50-cm intervals on June 20, 2010 (at the start of the rainy season, which was characterized by low rainfall, low soil water consumption, and low soil water content).

Soil depth	BL (%)	BSCs (%)	STBU (%)	STBU+BSCs (%)	CAKO (%)	CAKO+BSCs (%)
0–50 cm	11.7±0.0^a^	10.7±0.8^b^	9.0±1.1^c^	5.1±0.6^d^	10.5±0.0^b^	8.5±1.3^c^
50–100 cm	15.2±1.1^a^	15.4±1.1^a^	9.3±0.9^c^	9.9±1.2^c^	11.2±0.1^b^	9.6±1.3^c^
100–150 cm	15.4±0.0^b^	17.3±0.8^a^	12.1±0.8^c^	12.5±1.6^c^	11.8±1.0^c^	6.9±0.2^d^
150–200 cm	12.2±0.8^d^	12.8±0.7^c^	14.8±0.9^a^	13.5±0.8^b^	13.4±0.8^b^	7.2±0.0^e^
200–250 cm	13.0±0.0^c^	15.0±0.8^a^	14.2±0.9^ab^	14.4±1.0^a^	14.8±1.2^a^	13.4±0.9^c^
250–300 cm	13.9±0.2^d^	13.7±0.5^d^	16.0±1.2^b^	17.0±0.5^a^	15.8±1.3^b^	14.7±0.8^c^

Note: Different superscript letters in the same row indicate significant differences at *P*< 0.05

The soil water content of each 50-cm soil profile layer was determined as the mean of measurements at 10-cm intervals

BL, bare land; BSCs, biological soil crusts; STBU, *Stipa bungeana* Trin.; CAKO, *Caragana korshinskii* Kom.

**Table 5:** The soil water content in each treatment at 50-cm intervals on September 21, 2010 (at the end of the rainy season, which was characterized by high rainfall, high soil water consumption, and high soil water content).

Soil depth	BL (%)	BSCs (%)	STBU (%)	STBU+BSCs (%)	CAKO (%)	CAKO+BSCs (%)
0–50 cm	18.4±0.7^b^	20.3±1.9^a^	20.4±0.2^a^	14.1±0.8^e^	17.0±0.5^c^	16.1±1.8^d^
50–100 cm	17.4±1.5^a^	16.8±1.6^a^	11.7±1.5^b^	11.0±1.7^b^	10.4±0.1^bc^	9.0±1.7^d^
100–150 cm	14.8±0.6^a^	15.1±2.1^a^	14.0±1.6^b^	12.9±1.2^c^	9.8±0.3^d^	7.0±0.9^e^
150–200 cm	11.6±0.3^c^	12.2±0.9^c^	16.0±1.0^a^	14.9±0.5^b^	11.7±0.3^c^	7.3±0.5^d^
200–250 cm	13.3±0.0^c^	14.5±1.1^b^	16.4±1.2^a^	16.8±1.1^a^	13.2±1.0^c^	12.7±1.0^c^
250–300 cm	14.8±0.2^c^	14.3±0.2^cd^	17.2±1.5^a^	16.9±0.6^a^	15.9±1.2^b^	14.9±0.5^c^

Note: Different superscript letters in the same row indicate significant differences at *P*< 0.05

The soil water content of each 50-cm soil profile layer was determined as the mean of the measurements at 10-cm intervals

BL, bare land; BSCs, biological soil crusts; STBU, *Stipa bungeana* Trin.; CAKO, *Caragana korshinskii* Kom.

Early in the rainy season (Fig 5A), the soil moisture in the top 50 cm of soil in the BSC plots was lower than that in the BL plots (*p*<0.05, Table 4), and it was higher in the soil layers below 100 cm in the BSC plots than in the BL plots. At the end of the rainy season (Fig 6A), the soil moisture was higher in the BSC plots than in the BL plots in most soil layers (*p*<0.05, Table 5). The BSCs treatment had higher infiltration amounts (the efficiency of the increasing infiltration was 12.4%, Table 2). The soil layers from 0–100 cm were the most affected by BSCs.

Throughout the study period, the STBU+BSCs treatment (Figs 5B and 6B) had higher soil water levels at >150 cm depth in comparison with the BL plot (*p*<0.05, Tables 4 and 5), whereas these levels were lower than they were in the BL plot at depths of <150 cm. The increased infiltration in the STBU+BSCs treatment (infiltration increased by 21.9%, whereas BSC alone increased infiltration by 2.5%) was slightly higher than that of the STBU treatment. These results may indicate that the combined STBU+BSCs treatment had greater water consumption in the <150 cm soil layer. The soil moisture at different depths in the CAKO plot is shown in Figs 5C and 6C. These data also suggest that there was higher water consumption in the CAKO treatment because the soil moisture at <150 cm was significantly lower than that in the BL treatment (*p*<0.05, Tables 4 and 5).

## Discussion

### The retention and consumption of soil moisture

The hydrological effects of BSCs are extremely complex. Their effects on infiltration and runoff can be influenced by rainfall properties, BSC characteristics, topography, and soil type, and the effects of various factors may vary in strength under different circumstances [24]. Several similar discussions assume that BSCs reduce the soil infiltration rate, attenuate the soil infiltration capacity, and degrade the soil water regime [25,26]. These assumptions are only partially supported by our current results and previous studies [27,28]. Many studies have suggested that the lower infiltration rate caused by BSCs may reduce the infiltration capacity, increase runoff, and intensify erosion, especially in areas prone to water erosion [29,30,31]. Our previous study [27] also showed that BSCs alone significantly reduced the initial soil infiltration rate to 53% and reduced the stable soil infiltration rate to 52% compared with bare soil. However, the amount of accumulated infiltration from the present treatment plots was higher after each rainfall event, and the corresponding runoff was significantly lower than that of the BL plot. We conclude that a decrease in the infiltration rate does not always produce a decrease in the infiltration capacity. The presence of BSCs can refine the soil particles [32], reduce the void ratio [33,34], and reduce the infiltration rate. At the same time, the soil hygroscopicity can increase with the presence of BSCs, which can enhance the soil surface roughness [10] and substantially decrease the rate of runoff in water erosion regions [35]. Therefore, the presence of BSCs can extend the water residence time on sloped surfaces. The effects caused by a reduced infiltration rate can be offset and exceeded by an increased infiltration time, which can increase the infiltration capacity after each rainfall event.

The current study showed that BSCs may have increased the soil water consumption in the upper soil layer, with moss transpiration and evaporation through the capillary system associated with a compact layer of BSCs, especially when the soil was relatively dry. A previous evaporation experiment by Xiao et al. [26] showed that a lower level of soil moisture corresponded to greater evaporation under certain circumstances. This result is consistent with our findings, and it is probable that the observed effect is related to increased plant transpiration from the soil crust. Soil moisture absorption may also increase with the presence of BSCs [10], resulting in an increased infiltration amount. Consequently, infiltrated water could persist in the upper soil layer, producing higher rates of evaporation. At the end of the growth season, lower temperatures may have led to lower observed evaporation and transpiration rates than those occurring during the growing season. Correspondingly, the soil moisture in the top layers of the BSCs treatment was greater than that of the BL plots. This finding is consistent with the results of a previous study performed in the Tengger Desert in China [25], where the infiltration depth was reduced because of the higher water-holding capacity of the BSCs, thereby increasing the topsoil moisture.

In the current experiment, the soil moisture in most cases was significantly higher in the vegetation treatments without BSCs than in those with BSCs (a difference in the soil moisture was detected at the following depths: BSCs < 0.5 m, STBU+BSCs < 1.5 m, and CAKO+BSCs < 2.0 m) although the amount of infiltration in the latter was even slightly higher than that in the former (Table 2). These findings indicate that BSCs further exacerbated soil moisture when associated with vegetation. When vegetation and BSCs coexisted, the competition for water resources between the BSC and vegetation may have caused the vegetation to use more soil water from deeper soil layers, and BSCs primarily used water from the top layers. The fertilization effects of BSCs [15] likely promoted the growth of vegetation, which led to increased water uptake by the vegetation. This fertilization effect is supported by the finding that the influence of the combined treatments on the soil moisture level was less in deeper soil layers in comparison with that of the vegetation-only treatment. A comparison of the combined treatments showed that the BSC influence on soil moisture was much stronger in the presence of a shrub species (CAKO) than with a grass species (STBU), most likely because of the difference in the rooting depths of these plants. Therefore, we propose that the BSC effect on the soil moisture is not a direct effect, but is instead realized via competition with vegetation for water resources and/or the fertilization effect of BSCs that stimulates plant growth.

### Implications for disturbance and protection

Soil hydrology processes including infiltration, runoff, and evaporation and strongly depends on the crust type, soil characteristics, initial soil moisture, and rainfall intensity. Despite the complexity of these affecting factors, the runoff coefficient can be predicted over extended time scales based on the surface storage capacity [36]. Our study showed that BSCs play significant roles in reducing runoff and soil erosion during the rainy season. Soil erosion was reduced by 81% in the BSC treatment in comparison with the BL plots, and this effect was further strengthened when BSCs were combined with vegetation. These results were consistent with previous findings [17,18]. By contrast, the contribution of BSCs to erosion reduction was negligible in the combined BSC and vegetation treatments. For example, the contributions of BSCs to runoff reduction and soil loss in the STBU+BSCs plots were 7.4% and 0.7%, respectively, and 5.7% and 0.3% in the CAKO+BSCs plots, respectively. Furthermore, BSCs may influence soil moisture through competition with vegetation for water resources and fertilization effects, both of which would enhance water consumption from deeper soil layers by the plants.

Based on the effects of BSCs on soil erosion and soil moisture, we suggest that the long-term goal to support a sustainable environment can be achieved through an appropriate coverage with vegetation and BSCs. However, BSCs also have the ability to increase the infiltration capacity, reduce runoff and erosion, and facilitate plant growth through fertilization, although BSCs may compete with vegetation for water resources. Therefore, the removal disturbance of BSCs should be performed with extreme caution for the purpose of improving soil moisture levels. BSCs can be re-established rapidly, and their soil stabilization and erosion protection functions may be recovered within one rainy season after disturbance [37]. However, removing BSCs from plots with little or no vegetation cover on sloping land greatly increases the extent of soil erosion [31]. Such disturbance also significantly reduces the recharge from rainfall infiltration even though the water consumption in the surface layer is reduced slightly after removing the BSCs. From the perspective of soil and water conservation, the protection of BSCs in plots with less vegetation is of primary importance especially during the rainy season because of water erosion. The intermediate-disturbance hypothesis suggests that moderate disturbance levels will maximize the species diversity in BSCs [38]. If disturbances are deemed necessary, this work should be performed with care at the end of the rainy season to avoid severe erosion and water loss from the topsoil. For example, Li et al. (2009) proposed that low-density grazing disturbance is preferred for a sustainable ecological use [39].

It should be noted that our study was performed under simulated removal disturbance conditions. With different disturbance levels or methods, the BSCs may have different effects on erosion, infiltration, water consumption, and competition with vegetation. Sonia et al. [40] found that removing cyanobacteria-dominated or lichen-dominated BSCs led the soil moisture in the upper layer to decrease, which is inconsistent with our results likely because of the BSC type. Another report [41] demonstrated that crust removal increased water infiltration in other crust types except lichen and moss crusts on coarse-textured soil. In addition, the contradictory relations between plants and BSCs have been attributed to one or two independent processes of vascular plant population recruitment. It is necessary to understand the BSC effects by synthesizing available information at each stage, including germination, seedling survival, and seedling growth [22]. Exotic plant emergence has been found to be greater after BSC disturbance, and native plants were shown to be less likely to emerge [42]. Currently, the consequences that are aimed at different frequencies, times, and intensities of BSC disturbances such as fire, grazing, removal, and trampling are not completely understood [43].

From the analysis above, the disturbance views concluded from our experiment could not be extrapolated directly to a different region. To implement a rational disturbance as an effective management practice of BSC resources on the Loess Plateau, further studies should investigate the long-term effects of BSC and vegetation combinations on soil moisture and erosion [44]. More discussion about the manner (raking, removal, trampling, grazing, fire, etc.), intensity (light, moderate, or severe) and moment (rainy or drought season, growing or dormant period) of the disturbance must be conducted widely and deeply to determine the optimal BSC disturbance. Nevertheless, the current study demonstrates the contribution of BSCs to soil erosion reductions with and without the presence of vegetation. Our investigation reveals the combined effects of BSCs and vegetation regarding soil water utilization, thus implying that it is useful information for the effective management of BSCs.

## Conclusions

Our results demonstrate that BSCs alone significantly reduced runoff by 37.3% and soil loss by 81.0% and increased infiltration by 12.4% in comparison with BL. These crusts should be proposed for protection or development for the effective control of erosion where vegetation is sparse, although BSCs exacerbate the consumption of soil water in the upper layer, especially during the drought season. However, if combined with *Stipa bungeana* Trin. (STBU) or *Caragana korshinskii* Kom. (CAKO), BSCs only contributed runoff reductions of 7.4% and 5.7%, soil loss reductions of 0.7% and 0.3%, and infiltration increases of 2.5% and 1.9%, respectively. Considering these negligible contributions from BSCs to erosion control and the remaining exacerbated consumption of soil water in the profile, removal or raking disturbances of BSCs in combination with a high vegetation cover could be considered to improve soil moisture levels and positively benefit vegetation growth without significantly increasing erosion, which is necessary and feasible.

## Supporting Information

S1 Fig The BSCs pavement processes and a panoramic view of the experimental plots during the second rainy season. (DOC)
