# Supplementary figures and images for: The Combined Effects of Moss-Dominated Biocrusts and Vegetation on Erosion and Soil Moisture and Implications for Disturbance on the Loess Plateau, China
Source: PLoS One. 2015 May 20;10(5):e0127394. doi: 10.1371/journal.pone.0127394 (PMC4439065; doi:10.1371/journal.pone.0127394)

Figure S1

| 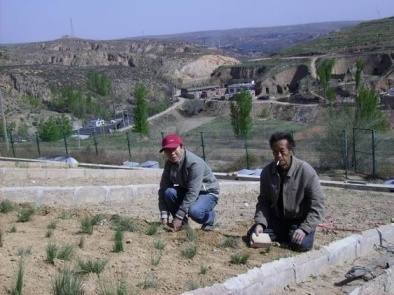  **STBU planting** | 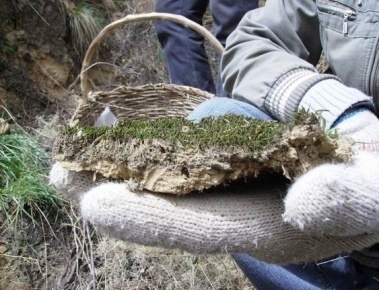  **Crust sampling** | 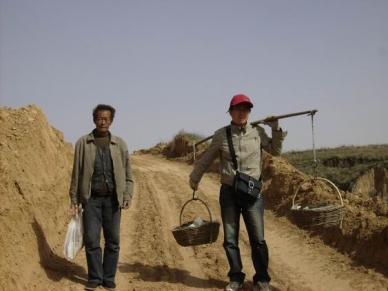  **Transporting samples** |
| --- | --- | --- |
| 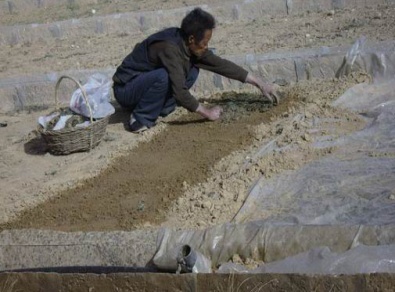  Crust paving | 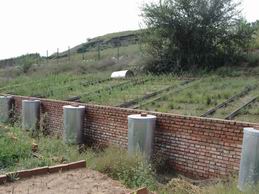 | 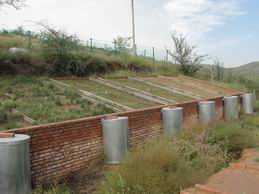 |

Supplement: S1 Fig — (DOC) [file pone.0127394.s001.doc]
